# Battling Influenza in Elderly Patients: A Case of Severe Influenza A With Complications in a High-Risk Patient

**DOI:** 10.7759/cureus.82303

**Published:** 2025-04-15

**Authors:** Argavan Ansari, Annie Taffaro, Zachary McSween, Jenny Lu, Alice Huang, Roxana Lazarescu

**Affiliations:** 1 Internal Medicine, Touro College of Osteopathic Medicine, New York City, USA; 2 Internal Medicine, Wyckoff Heights Medical Center, Brooklyn, USA; 3 Medicine, Touro College of Osteopathic Medicine, New York City, USA; 4 Internal Medicine, Wyckoff Heights Medical Center, New York City, USA

**Keywords:** at-risk-population, hypoxic respiratory failure, influenza, pneumonia, preventive measures, septic shock, vaccination

## Abstract

Influenza is a significant cause of morbidity and mortality in the elderly, particularly in those with chronic comorbidities and age-related immunosenescence. This case report details an 80-year-old male with a history of hyperlipidemia, diabetes mellitus, and dementia who developed a severe influenza A infection complicated by sepsis and hypoxic respiratory failure. Upon admission, the patient presented with fever, cough, and weakness, and was initially treated for viral pneumonia. Despite negative blood and urine cultures, influenza A was confirmed via reverse transcription polymerase chain reaction (RT-PCR). The patient developed hypotension, was unresponsive to fluid resuscitation, and was transferred to the ICU for pressor support with norepinephrine and albumin.

During his intensive care unit (ICU) stay, septic shock was managed with norepinephrine, with a target mean arterial pressure (MAP) of >65 mmHg. The patient also received respiratory support, initially on a 10L high-flow nasal cannula, and was later downgraded to a 4L nasal cannula. A sacral pressure ulcer and MRSA colonization further complicated his hospital course. Antibiotic therapy included ceftriaxone, azithromycin, and vancomycin, and the patient’s condition gradually improved, allowing for transfer to the intermediate care unit (IMCU) after seven days. This case underscores the importance of early antiviral therapy, the need for vigilant monitoring for secondary infections, and timely intervention to manage complications such as sepsis in elderly patients. Enhanced vaccination coverage and preventive measures are critical for reducing the incidence of severe influenza and associated complications in at-risk populations.

## Introduction

Influenza is a leading cause of morbidity and mortality worldwide, especially in vulnerable populations such as the elderly. The elderly are at increased risk due to age-related immunosenescence and chronic comorbidities, which often complicate the clinical course of the infection. Each year in the United States, severe cases of influenza and related complications result in an estimated 140,000 to 710,000 hospitalizations, with the majority affecting adults aged 65 and older [1,2]. The economic burden of influenza is significant, with costs mainly arising from hospitalization, medical care, and lost productivity. For those aged 65 and older, the direct and indirect costs associated with influenza are considerably higher when compared to younger populations [1,2].

Flu vaccination is vital for individuals aged 65 and older since they are at higher risk for severe complications, such as pneumonia, sepsis, and respiratory failure. For this age group, high-dose inactivated flu vaccines, such as high-dose quadrivalent influenza vaccine (Fluzone high-dose), adjuvanted inactivated influenza vaccine (Fluad), and recombinant influenza vaccine (Flublok), are generally recommended to boost immune response [3]. However, vaccine effectiveness is often lower in this group due to diminished humoral and cellular immune responses [2,4].

Pneumonia is a common complication in adults hospitalized with laboratory-confirmed influenza and significantly worsens clinical outcomes in these patients [1,2]. The development of pneumonia, particularly when it occurs as a secondary complication of influenza, increases the risk of prolonged hospital stays, mechanical ventilation, and even death. One of the major concerns with influenza-associated pneumonia is the development of secondary complications such as sepsis and acute respiratory failure, which can further raise the risk of mortality. A large study by Tenforde et al. found that patients treated with antivirals on the first day of hospitalization had the best outcomes, with a significantly lower mortality rate than those treated later [4]. When antiviral treatment is delayed, particularly in those with pneumonia, outcomes tend to worsen, with septic shock, respiratory failure, and higher mortality rates often occurring [4].

About one in 10 older adults hospitalized with influenza will die from their illness, and those who survive often experience poor outcomes after hospital discharge, including persistent diminished functional status and need for readmission [2]. The long-term consequences and increased need for healthcare utilization add to the overall economic burden on the healthcare system. The same multi-season study from 2012 to 2019 showed that patients who received antiviral treatment on days 2-5 had significantly higher 30-day all-cause mortality compared to those treated on day 0 [4]. This highlights the crucial importance of early testing and prompt treatment, especially in elderly patients who may exhibit atypical symptoms.

Annual vaccination remains the cornerstone for the prevention of influenza illness; however, because of age-related immune decline, standard influenza vaccines often do not provide sufficient protection in older adults. Two influenza vaccines approved in the United States for adults aged ≥65 years were developed with features aimed to boost immune responses in older adults: high-dose inactivated influenza vaccine and MF59-adjuvanted inactivated vaccine [2,4]. These vaccines trigger stronger antibody responses compared to standard influenza vaccines and have been associated with better protection against influenza-associated outcomes in several studies [4-7].

While observational studies have evaluated the benefits of enhanced vaccines when compared with standard vaccines for preventing influenza-associated hospitalizations, few studies have directly compared these enhanced vaccines to one another [2,5]. Considering the high economic burden of influenza, particularly in older adults, strategies like using enhanced vaccines may prove to be cost-effective by preventing severe disease, reducing hospitalizations, and lowering overall healthcare costs [1,5]. This case report aims to highlight the importance of early antiviral treatment and the monitoring of secondary complications like sepsis, to reduce the morbidity and mortality associated with severe influenza infections in elderly individuals.

## Case presentation

An 80-year-old male with a medical history of hyperlipidemia, type 2 diabetes mellitus, and dementia (baseline alert and oriented ×2) presented to the emergency department with fever, persistent cough, generalized weakness, poor oral intake, and non-bloody diarrhea of several days' duration. His family reported increasing lethargy and decreased responsiveness. On presentation, vital signs revealed a temperature of 101.8°F, hypotension with a mean arterial pressure (MAP) <50 mmHg, tachycardia (HR 110 bpm), and oxygen saturation of 89% on room air.

Initial labs showed elevated lactate (4.1 mmol/L), leukocytosis, and hyperglycemia. A chest x-ray demonstrated increased opacity in the left lower hemithorax, suggestive of a pleural effusion or infiltrate, correlating with pneumonia or an inflammatory process. Blood and urine cultures were obtained, and empiric treatment for presumed viral pneumonia with a possible bacterial superinfection was initiated. Influenza A was confirmed via nasopharyngeal swab reverse transcription polymerase chain reaction (RT-PCR) testing. Testing for *Legionella *and *Streptococcus pneumoniae* antigens was negative, as were initial blood and urine cultures.

Despite receiving an initial IV fluid bolus of 750 mL, the patient remained hypotensive and unresponsive to fluid resuscitation, prompting transfer to the intensive care unit (ICU) for vasopressor support. Norepinephrine was started peripherally and titrated to maintain MAP >65 mmHg. Concurrently, 5% albumin was administered to support oncotic pressure and improve intravascular volume status.

The patient developed acute hypoxic respiratory failure requiring high-flow nasal cannula (HFNC) oxygen therapy at 10 L/min with 60% FiO₂. Over several days, his oxygen requirements gradually decreased, and he was weaned to a 4 L/min nasal cannula by ICU day 6. While in the ICU, the patient tested positive for methicillin-resistant *Staphylococcus aureus* (MRSA) colonization via nasal swab. He was empirically treated with intravenous vancomycin in addition to ceftriaxone and azithromycin, as part of a broad-spectrum coverage protocol for possible secondary bacterial pneumonia, consistent with current sepsis guidelines [1].

On ICU day 4, the patient developed a stage 1 sacral pressure injury with extension to the bilateral gluteal region. A wound care consult was obtained. While detailed debridement or topical treatment documentation was not available in the chart, supportive care included repositioning, nutritional optimization with Pro-Stat protein supplement (Pro-Stat, Rockville, MD), and skin barrier protection. No signs of secondary wound infection were noted during his stay.

By ICU day 6, the patient’s hemodynamics had stabilized, and norepinephrine was discontinued following a transition to oral midodrine (15 mg every eight hours). His nutritional status improved with a carbohydrate-controlled puréed diet and multivitamin supplementation. His oxygenation continued to improve, and he was maintained on 3 L/min nasal cannula. He was subsequently downgraded to the intermediate care unit (IMCU) to complete his antibiotic therapy and receive ongoing wound care. On day 7, midodrine was tapered to 10 mg every eight hours, and his vital signs remained stable.

By hospital day 8, the patient had resolved septic shock, improving respiratory status, and stable hemodynamics without vasopressor support. He was considered medically stable for discharge. Discharge planning included outpatient wound care follow-up, primary care coordination, and physical therapy evaluation. Notably, the patient’s influenza vaccination status could not be verified, as documentation was unavailable in the electronic medical record at the time of admission or discharge.

**Figure 1:**
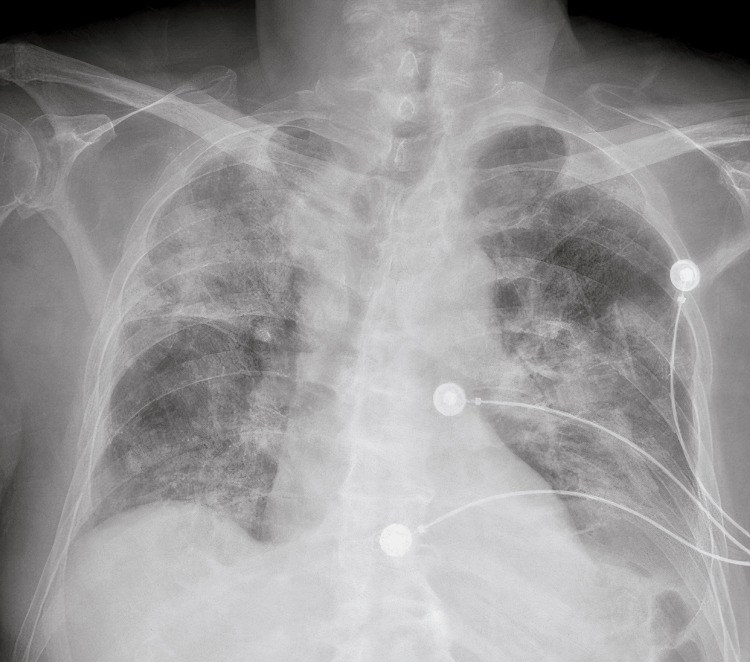
Chest x-ray (admission): increased opacity in the lower left side of the chest (hemithorax), consistent with either a pleural effusion or a pulmonary infiltrate.

**Figure 2:**
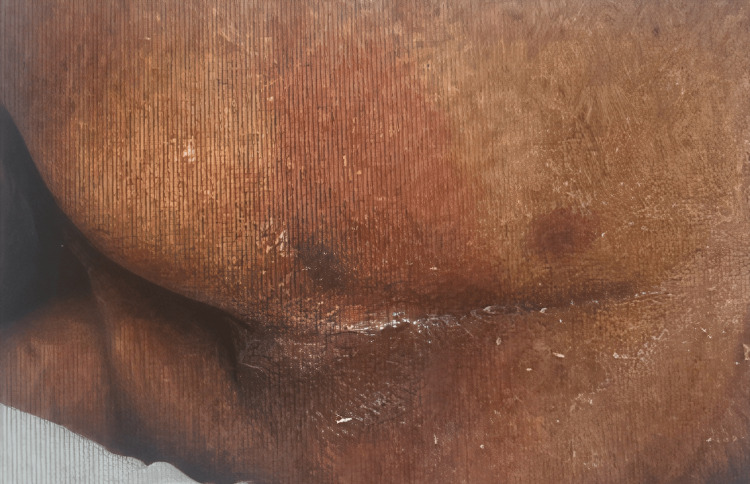
Wound 1: Sacral wound care for a stage 1 pressure injury on day 4 in the intensive care unit (ICU).

## Discussion

This case underscores the critical importance of early recognition and timely management of severe influenza complications in elderly patients with multiple comorbidities. The clinical course of this 80-year-old male illustrates how quickly influenza A can progress to life-threatening conditions such as septic shock and hypoxic respiratory failure, particularly in high-risk individuals with underlying conditions like diabetes mellitus, dementia, and hyperlipidemia. These comorbidities are known to impair immune responses and increase susceptibility to infection-related complications [1,2].

Evidence consistently supports the notion that early antiviral treatment significantly improves outcomes in hospitalized patients with influenza. A large multi-year analysis by Tenforde et al. found that initiating antiviral therapy on the first day of admission was associated with a substantially lower risk of mortality compared to delayed treatment on days 2-5 [4]. In this case, despite appropriate ICU management including vasopressors, albumin, and high-flow oxygen therapy, the patient’s condition may have benefited from even earlier antiviral initiation, though treatment timing was not clearly documented in the chart. Delayed recognition and access to care remain major barriers in geriatric populations, who may present atypically or rely on caregivers for symptom reporting [2,4].

The patient's development of MRSA colonization during his ICU stay is not uncommon in prolonged hospitalizations. Colonization without invasive infection still warrants close surveillance, and, in many institutions, empiric antibiotic coverage is broadened until culture results confirm the absence of active infection. The use of vancomycin in this case reflects a cautious approach, aligned with national guidelines on the empirical management of pneumonia in critically ill patients [1]. However, this also highlights the growing concern around antimicrobial stewardship in elderly patients with declining renal reserve and heightened risk for drug toxicity.

Compounding the clinical course, the patient developed a stage 1 sacral pressure injury with gluteal extension - a common yet preventable complication in patients with prolonged immobility and critical illness. While full wound care details were not documented, pressure ulcers are well-recognized markers of quality of care and predictors of poor long-term outcomes, particularly in older adults with dementia [2]. Nutritional support and frequent repositioning are fundamental components of pressure injury prevention and were appropriately integrated into this patient's care plan.

From a public health perspective, this case illustrates the profound value of preventive strategies, particularly vaccination. The patient’s influenza vaccination status was undocumented - a notable gap, given his age and risk profile. A study by Liang et al. showed that influenza vaccination reduced the risk of hospitalization for community-acquired pneumonia (CAP) by 28% among older adults during years with good vaccine strain matching [8]. Importantly, this benefit diminished in patients with high comorbidity scores, such as the subject of this case, underscoring the need for enhanced vaccines that offer stronger immunogenicity in elderly populations [4,5].

Furthermore, low testing rates for influenza in patients with CAP remain a widespread issue. Deshpande et al. reported that only 23.3% of patients hospitalized with CAP were tested for influenza, and just 11.5% of those tested returned positive results. Those who received antiviral treatment on hospital day 1 had lower mortality, shorter hospital stays, and reduced overall costs compared to those treated later or not at all [7,8]. This finding reinforces the importance of incorporating influenza testing as a routine diagnostic step in elderly patients presenting with respiratory symptoms, particularly during flu season.

Finally, this case reiterates that influenza-related complications often extend beyond acute hospitalization. Elderly survivors of severe influenza frequently experience lingering functional decline, malnutrition, and high rates of readmission [2,9]. A multidisciplinary approach, involving infectious disease specialists, intensivists, wound care nurses, nutritionists, and physical therapists, is essential to address both acute management and the long-term sequelae of influenza in older adults.

## Conclusions

This case underscores the complex challenges of managing severe influenza in elderly patients, particularly those with underlying comorbidities. The 80-year-old male developed complications such as hypoxic respiratory failure, septic shock, and secondary MRSA colonization, which reflect the rapid deterioration often seen in older adults due to immunosenescence and reduced physiologic reserves. Despite prompt hospitalization and aggressive interventions - including vasopressors, high-flow oxygen, broad-spectrum antibiotics, and nutritional support - he endured a prolonged hospital stay complicated by a pressure ulcer. These outcomes highlight the broader vulnerabilities of geriatric care, where complications often lead to long-term functional decline, institutionalization, and rehospitalization. The case also emphasizes the importance of early antiviral therapy, which, if initiated within 48 hours of symptom onset, can significantly reduce mortality and ICU admissions. Delayed treatment - often due to atypical presentations or caregiver-dependent reporting - limits this window of opportunity, making timely diagnosis and intervention critical.

Furthermore, this case illustrates systemic gaps in prevention and care coordination for high-risk elderly populations. The lack of documented influenza vaccination points to a missed opportunity for prevention, particularly when enhanced vaccines like high-dose or adjuvanted formulations have proven efficacy in older adults. Routine assessment and documentation of vaccination status should be embedded across all healthcare settings, especially during flu season. Rapid molecular testing for influenza should be prioritized for elderly patients presenting with respiratory symptoms, even when bacterial infections are suspected. Beyond pharmacologic measures, multidisciplinary approaches involving infectious disease specialists, wound care teams, nutritionists, and social workers are vital to ensuring comprehensive care. Prevention of complications, such as pressure ulcers, through ICU protocols and mobilization strategies must also be emphasized. Ultimately, this case serves as a reminder that while supportive care can improve survival, robust preventative strategies - especially vaccination and early treatment - are key to reducing influenza-related morbidity, mortality, and healthcare utilization in geriatric populations.
